# Supplementary material for: The Systems Biology Research Tool: evolvable open-source software
Source: BMC Syst Biol. 2008 Jun 29;2:55. doi: 10.1186/1752-0509-2-55 (PMC2446383; doi:10.1186/1752-0509-2-55)
Supplement: Additional file 1 — SBRT Archive. An archive of the current version of the Systems Biology Research Tool. [file 1752-0509-2-55-S1.zip › sbrt-1.4.0/doc/developers_guide/api/sbrt/kernel/math/optimization/QuadraticProgramSolver.html]

QuadraticProgramSolver


|  |  |  |
| --- | --- | --- |
| **Overview** \| **Package** \| **Class** \| **Use** \| **Tree** \| **Deprecated** \| **Index** \| **Help** | |  |
| **PREV CLASS**   NEXT CLASS | **FRAMES**    **NO FRAMES**     **All Classes** |  |
| SUMMARY: NESTED \| FIELD \| CONSTR \| METHOD | DETAIL: FIELD \| CONSTR \| METHOD |  |


---


## sbrt.kernel.math.optimization Interface QuadraticProgramSolver<R,C>

**Type Parameters:**: `R` - the row variable type.: `C` - the column variable type.

**All Superinterfaces:**: ProgramSolver<R,C>

**All Known Implementing Classes:**: CplexSolver

---

``` public interface QuadraticProgramSolver<R,C> extends ProgramSolver<R,C> ```

This interface is used to represent quadratic program solvers.

**Author:**
:   This interface was written and documented by
    Jeremiah Wright while in the Wagner lab.

---

| **Method Summary** | |
| --- | --- |
| `void` | `setConstraint(QuadraticExpr<? extends C> expression, Interval c)`             Sets a constraint on the provided quadratic expression of column variables. |
| `void` | `setObjectiveFunction(QuadraticExpr<? extends C> expression)`             Sets the objective function to the provided quadratic expression of column variables. |
| `void` | `setObjectiveFunction(QuadraticExpr<? extends C> expression, OptimizationSense sense)`             Sets the objective function and optimization sense to the provided values. |

**Methods inherited from interface sbrt.kernel.math.optimization.ProgramSolver**

`clear, getObjectiveValue, getSolutionVector, getStatus, isColumnVar, isRowVar, optimize, setColumnConstraint, setColumnConstraints, setConstraint, setConstraints, setMatrixElement, setObjectiveFunction, setObjectiveFunction, setOptimizationSense, setParamFile, setRowConstraint, setRowConstraints`

**Method Detail**

### setConstraint

```
void setConstraint(QuadraticExpr<? extends C> expression,
                   Interval c)
```

:   Sets a constraint on the provided quadratic expression of
    column variables.

    :   **Parameters:**: `expression` - a linear combination of column variables for which the constraint is set.: `c` - the constraint.

---


### setObjectiveFunction

```
void setObjectiveFunction(QuadraticExpr<? extends C> expression,
                          OptimizationSense sense)
```

:   Sets the objective function and optimization sense to the provided
    values.

    :   **Parameters:**: `expression` - a linear combination of column variables from this program solver.: `sense` - the optimization sense. **See Also:**: `setObjectiveFunction(QuadraticExpr)`, `ProgramSolver.setOptimizationSense(OptimizationSense)`

---


### setObjectiveFunction

```
void setObjectiveFunction(QuadraticExpr<? extends C> expression)
```

:   Sets the objective function to the provided quadratic expression of
    column variables.

    :   **Parameters:**: `expression` - a quadratic expression of column variables from this program solver.


---


|  |  |  |
| --- | --- | --- |
| **Overview** \| **Package** \| **Class** \| **Use** \| **Tree** \| **Deprecated** \| **Index** \| **Help** | |  |
| **PREV CLASS**   NEXT CLASS | **FRAMES**    **NO FRAMES**     **All Classes** |  |
| SUMMARY: NESTED \| FIELD \| CONSTR \| METHOD | DETAIL: FIELD \| CONSTR \| METHOD |  |


---
